# The effect of bovine colostrum supplementation on intestinal injury and circulating intestinal bacterial DNA following exercise in the heat

**DOI:** 10.1007/s00394-018-1670-9

**Published:** 2018-03-24

**Authors:** Daniel S. March, Arwel W. Jones, Rhys Thatcher, Glen Davison

**Affiliations:** 1 Department of Infection, Immunity and Inflammation, College of Life Sciences, University of Leicester, Leicester, UK; 2 Lincoln Institute for Health, University of Lincoln, Lincoln, UK; 3 Institute of Biological, Environmental and Rural Sciences, Aberystwyth University, Aberystwyth, UK; 4 Endurance Research Group, School of Sport and Exercise Sciences, University of Kent, Medway Campus, Chatham Maritime, ME4 4AG UK

**Keywords:** Intestinal fatty acid-binding protein, Cellular injury, Strenuous exercise, Bacterial translocation, Intestinal permeability, Core temperature, Environment

## Abstract

**Purpose:**

Exercise-induced changes in intestinal permeability are exacerbated in the heat. The aim of this study was to determine the effect of 14 days of bovine colostrum (Col) supplementation on intestinal cell damage (plasma intestinal fatty acid-binding protein, I-FABP) and bacterial translocation (plasma bacterial DNA) following exercise in the heat.

**Methods:**

In a double-blind, placebo-controlled, crossover design, 12 males completed two experimental arms (14 days of 20 g/day supplementation with Col or placebo, Plac) consisting of 60 min treadmill running at 70% maximal aerobic capacity (30 °C, 60% relative humidity). Blood samples were collected pre-exercise (Pre-Ex), post-exercise (Post-Ex) and 1 h post-exercise (1 h Post-Ex) to determine plasma I-FABP concentration, and bacterial DNA (for an abundant gut species, Bacteroides).

**Results:**

Two-way repeated measures ANOVA revealed an arm × time interaction for I-FABP (*P* = 0.005, with greater Post-Ex increase in Plac than Col, *P* = 0.01: Plac 407 ± 194% of Pre-Ex vs Col, 311 ± 134%) and 1 h Post-Ex (*P* = 0.036: Plac 265 ± 80% of Pre-Ex vs Col, 229 ± 56%). There was no interaction (*P* = 0.904) but there was a main effect of arm (*P* = 0.046) for plasma Bacteroides/total bacterial DNA, with lower overall levels evident in Col.

**Conclusion:**

This is the first investigation to demonstrate that Col can be effective at reducing intestinal injury following exercise in the heat, but exercise responses (temporal pattern) of bacterial DNA were not influenced by Col (although overall levels may be lower).

## Introduction

Increases in core temperature, and heat stress along with intestinal hypoperfusion, and ischaemia–reperfusion (I–R), contribute to intestinal injury and changes in permeability during exercise [1–7]. These physiological responses are likely exacerbated when exercising in hot and humid environments. Moreover, exercise in the heat is both common and necessary in many sport (for athletes) or work contexts (e.g. military, firefighters). However, under extreme circumstances exhaustive exercise in such environments can result in severe endotoxaemia (the translocation of bacterial lipopolysaccharide (LPS) into the central circulation) [8, 9], through intestinal injury and changes in permeability, which is thought to be associated with acute inflammation, sepsis, shock and organ failure that in rare cases may be fatal.

Nutritional interventions to maintain the integrity of the intestinal barrier and, therefore, avoiding these complications during and following exercise in the heat are somewhat limited. However, bovine colostrum (Col) has shown to be both effective in blunting the heat-induced increase in permeability in vitro and in vivo in animals and humans [1, 2, 10, 11]. Previously Col has blunted both the exercise-induced increase in intestinal permeability and circulating intestinal fatty acid-binding protein (I-FABP) (a marker of intestinal cellular injury) in the exercise stress model in humans where an increase of ~ 1.5 °C in core temperature was observed [1, 2, 11].

It is important to note, however, that this increase (following 20 min of running at 80% ${\dot {\text{V}}}{\text{O}_{2\text{peak}}}$) may be relatively mild considering that strenuous athletic events in the heat can evoke much higher elevations in core temperatures [12, 13]. The implications of changes to the intestinal barrier during exercise and heat is an issue of increased significance as there is a global increase in ambient temperatures during athletic competition [14].

We have previously shown that changes in plasma I-FABP immediately following treadmill running occur concurrently (and correlate) with changes in intestinal permeability as measured by the 5-h urinary excretion ratio between lactulose and rhamnose (L/R) [2], and also that both are blunted by Col supplementation. However, a recent study [15] has indicated that when core temperature is elevated above 39 °C during exercise then Col may not be effective in blunting the increase in plasma I-FABP. Although, in this study [15] there were variable running times between the two conditions [placebo (Plac) and Col], imposing different stresses that may have compromised the validity of these results.

Some previous investigations have quantified changes in circulating LPS following exercise [16, 17] to indicate changes in intestinal permeability. However, there are issues pertaining to the collection and analysis of blood samples [through the limulus amoebocyte lysate assay (LAL)], in addition to the detection of LPS, suggesting that this marker may not be ideal [18, 19]. Some clinical studies have measured intestinal-derived circulating bacterial DNA via 16S rDNA PCR assays to indicate changes in intestinal permeability [19–22], which has been suggested to be more specific to targeted bacterial strains and can overcome some of the sensitivity and specificity issues that exist for the LPS LAL assays. However, circulating bacterial DNA (as a marker of bacterial translocation) has not previously been assessed within an exercise and heat stress model.

Therefore, the primary aim of this study is to determine whether 14 days of oral Col supplementation can blunt the heat- and exercise-induced increase in intestinal injury measured by plasma I-FABP. The secondary aim was to determine the effects on circulating bacterial DNA, as a marker of bacterial translocation. It is hypothesised that exercise and heat would present a larger challenge to intestinal integrity (greater increase in plasma I-FABP) and intestinal permeability than we observed in exercise alone in our previous work [2], resulting in a large increase in this injury marker and circulating bacterial DNA. However, it is hypothesised that Col supplementation would blunt these increases.

## Methods

### Ethical approval

The study was conducted in accordance with the Declaration of Helsinki and approved by the Aberystwyth University Research Ethics Committee. All participants were informed both verbally and in writing of the nature and risks of the experimental procedures after which written consent was obtained at least 7 days prior to the preliminary visit.

### Sample size

Based on our previous observed [2] post-exercise change (compared to pre-exercise) in plasma I-FABP with 18 participants (no dropouts) (297 ± 285 pg/mL for Plac and 6 ± 283 pg/mL for Col), we calculated (G*Power version 3.1.9.1, Kiel, Germany) that *n* = 12 was required to detect a significant difference between Plac and Col arms with 95% power and alpha set at 0.05.

### Participants

Twelve healthy male participants who were all regular exercisers participated in this study (mean ± SD: age 26 ± 6 years; body mass 79.5 ± 9.4 kg; height 180 ± 7 cm; body mass index 24 ± 2 kg m^−2^; ${\dot {\text{V}}}{\text{O}_{2\text{peak}}}$ 55.8 ± 4.8 mL kg^−1^ min^−1^; peak speed in ramp test 18.2 ± 0.8 km h^−1^; running speed at 70% ${\dot {\text{V}}}{\text{O}_{2\text{peak}}}$ 11.0 ± 0.6 km h^−1^). All trials took place between October 2013 and March 2014 in the United Kingdom. The range for average air temperature measured at a local climate station (25 miles from our laboratory) for these months has been reported as 3.8–14.8 °C (http://www.metoffice.gov.uk).

### Inclusion/exclusion criteria

Inclusion criteria were: age 18–45 years, free of illness symptoms for 4 weeks prior to the study, and no non-steroidal anti-inflammatory drug consumption or other use (e.g. via over the counter creams) for 4 weeks prior to the study. Individuals were unable to participate if they were currently a smoker, had any history of gastrointestinal disorders or surgery, or were allergic or intolerant to dairy products. Participants were also excluded if they were not able to complete an exercise test that required maximum effort or had recent history (< 6 months) of supplement use or a history of previous heat injury/heat stroke. All participants were permanent residents in the United Kingdom for at least 8 weeks before beginning the study.

### Study design and nutritional intervention

**Fig. 1:**
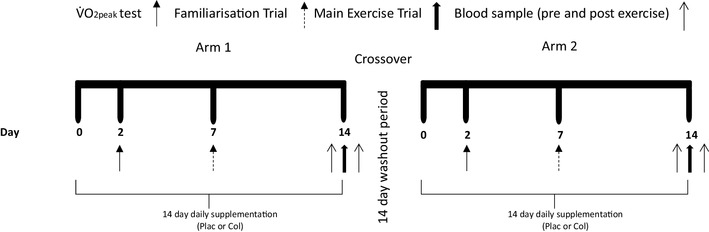
Schematic of study design. Each participant took part in a double-blind, placebo-controlled, randomised, crossover study. Participants received oral supplementation with 20 g day^−1^ of either Plac or Col for 14 days with a 14-day washout period between study arms. After the 14-day washout these procedures were repeated with participants supplementing with the opposite-test substance (Plac or Col)

In this double-blind, placebo-controlled, randomised, crossover study, participants were required to undertake exercise under two conditions (study arms): Plac supplementation or Col with a minimum ‘washout period’ of 2 weeks between arms (Fig. 1). We have previously shown that a ‘washout’ period of 2 weeks is sufficient for Col and there are no carry-over effects for plasma I-FABP [2], or other markers of intestinal permeability [2, 11, 23]. For each arm, participants performed one preliminary visit (determination of running speed at 70% ${\dot {\text{V}}}{\text{O}_{2\text{peak}}}$), one familiarisation and one main trial over an approximate 2-week period. Participants supplemented with 20 g day^−1^ (10 g prior to morning and evening meal) of either Plac or Col for 14 days, which was administered in a randomised crossover fashion using an online randomisation tool (http://www.randomization.com). Four participants’ first-test substance was Plac whilst eight ingested Col as the first-test substance. Two days into the supplementation period participants performed a trial to determine running speed at 70% ${\dot {\text{V}}}{\text{O}_{2\text{peak}}}$. Seven days into the supplementation period participants performed the familiarisation trial. Seven days later participants performed the main trial. After at least 2 weeks of washout, these procedures were repeated with participants supplementing with the opposite test substance (Plac or Col). Supplements were supplied in sealed tubes labelled as ‘A’ and ‘B’ by an independent investigator who was not involved in the data collection or analysis. Investigators were unblinded following the completion of data collection and analysis. Participants completed a 24-h food diary on the day before the first exercise visit and repeated this diet for the subsequent exercise visit in the opposing arm. Participants were instructed to avoid alcohol consumption and strenuous activity or exercise for 2 days before each preliminary and main exercise visit.

### Bovine colostrum

The Col was supplied by Neovite UK as used in previous studies from our laboratory [2, 11]. The approximate energy content was 311.5 kJ (74.4 kcal) per 20 g [i.e. 80% protein, 9% carbohydrate (lactose), 1.8% fat]. The placebo was an isoenergetic and isomacronutrient milk protein concentrate and was indistinguishable in terms of appearance and taste from the Col powder.

### Preliminary visits

Participants reported to the laboratory, and body mass and stature were recorded. Body mass was recorded using laboratory scales (Seca 899, Seca GmbH & Co, Hamburg, Germany) with the participants unshod and wearing the same clothing as during the experimental trials (typically lightweight shorts and t-shirt). Following this, participants completed a graded exercise treadmill test (Woodway Ergo-Line Treadmill PPS55i Med, Woodway GmbH, Weil am Rhein, Germany) to voluntary exhaustion with the grade set at 1% [24], as previously described [25].

### Familiarisation trial

A familiarisation trial took place 7 days before the main trial to ensure that all participants were accustomed to the testing procedure and ensure that the correct exercise intensity had been identified from the preliminary trial. Participants performed 60 min of treadmill running at 70% ${\dot {\text{V}}}{\text{O}_{2\text{peak}}}$. Core body temperature, heart rate (HR), rating of perceived exertion (RPE) [26], and thermal sensation were recorded using a 21-point scale ranging from − 10 (cold impossible to bear) to + 10 (heat impossible to bear) (used previously in our laboratory during nutritional studies of exercise in the heat by Tumility et al. [27, 28]) every 10 min during the trial. Fluid (ordinary tap water) was provided to participants at a rate of 2.5 mL kg body mass^−1^ every 20 min during exercise. The purpose of this trial was to habituate the participants to the physiological measurements (mentioned above) and to exercising in the heat.

### Main trial

Following an overnight fast, participants consumed 500 mL of ordinary tap water 1 h before arriving at the laboratory at 07:00, and confirmed verbally that they had adhered to the supplement intake instructions and other study requirements. Participants were then asked to remain seated for 10 min before a blood sample was drawn (Pre-Ex) (less than 30 s of stasis) from the antecubital vein using a 21-gauge precision needle (Becton–Dickinson, Oxford, UK) and collected into vacutainer tubes (6 mL into K_2_EDTA-treated tube and 6 mL into a heparin-treated tube, BD Vacutainer Systems, Plymouth, UK). Following this participants provided a urine sample (Pre-Ex), and the volume was recorded before an aliquot was removed and immediately frozen at − 80 °C for later measurement of osmolality using freezing point depression (CV for urine osmolality 4%) (Osmomat 030, Gonotec, Berlin, Germany). A rectal thermistor (Grant Instruments, Cambridge, England) was self-positioned by each participant 10 cm beyond the anal sphincter to enable core temperature measurement and a short-range heart rate telemetry band was fitted (Polar S610i, Polar Electro Oy, Tampere, Finland). Participants then entered the climatic chamber (Design Environmental, Gwent, Wales, UK) which was maintained at 30.0 ± 0.1 °C, and 60 ± 0% relative humidity (monitored every 20 min) and placed their feet either side of the treadmill belt until the relevant speed was attained. They were then instructed to begin running at a constant speed equivalent to 70% ${\dot {\text{V}}}{\text{O}_{2\text{peak}}}$ on a motorised treadmill with a 1% grade [24] for 60 min or until core temperature reached 40 °C (whichever came first). Fluid (ordinary tap water) was provided to participants at a rate of 2.5 mL kg body mass^−1^ every 20 min during exercise. HR, RPE, and thermal sensation were recorded using the scales previously described every 10 min during the trial. Core body temperature was also recorded every 10 min during the trial and then again 5 min following the cessation of exercise (the change between pre-exercise and peak, which was always 5 min post, was calculated). The mean of the physiological responses were calculated to ensure a similar physical stress was imposed between arms. Following running, participants were instructed to support their weight with their hands on the hand rails and place their feet either side of the treadmill belt. Participants were then immediately removed from the climatic chamber and seated outside and a further venous blood sample (Post-Ex) was obtained. A maximum of 3 min elapsed between end of exercise and this blood sample being drawn. Participants then remained in the laboratory until 1 h post-exercise when a further (1 h Post-Ex) venous blood sample and urine sample were obtained.

### Blood analysis

Haemoglobin (Hb) was determined on EDTA-treated blood samples using an automated haematology analyser (ABX Pentra 60C+, Horiba Medical, Montpellier, France). Blood lactate and blood glucose (from the heparin-treated tube) were determined using an automated analyser (YSI 2300 Stat Plus, Yellow Springs, OH, USA), and haematocrit was determined by standard microcentrifugation (Micro Haematocrit Mk5 Centrifuge, Hawkesley, Lancing, UK). Haematocrit and Hb concentration were used to estimate post-exercise changes in blood and plasma volume (compared to the Pre-Ex sample) using the equations of Dill and Costill [29]. The blood sample collected in the K_2_EDTA-treated tube was centrifuged at 1500×*g* for 10 min at 4 °C. The plasma from the K_2_EDTA-treated tube was pipetted into polypropylene microcentrifuge tubes (Eppendorf, Hamburg, Germany) before being stored at − 80 °C for later analysis (plasma I-FABP).

### Plasma I-FABP analyses

Plasma I-FABP was determined in duplicates via an ELISA kit (Hycult, Biotechnology, Uden, The Netherlands) according to the manufacturer’s instructions. Sterile procedures were observed throughout the assay. Inter-assay CV was 6.9%. As a result of large inter-participant variability in plasma I-FABP, we [2], and other groups [30–32], have previously reported plasma I-FABP as both an absolute change (in pg/mL) and a percentage change, compared to pre-exercise. To be consistent with these previous reports we have once again presented changes in this marker as both percentage and absolute values.

### Plasma bacterial DNA

Bacterial DNA was detected using a quantitative real-time polymerase chain reaction assay (qPCR) on a LightCycler 96 instrument (Roche, Basel, Switzerland). DNA was isolated from plasma using a QIAamp DNA blood mini kit (QIAGEN GmbH, Hilden, Germany) in accordance with the manufacturer’s instructions. Total bacterial DNA was quantified by the methods of Zhu et al. [33] using a Universal probe library probe (Roche, Basel, Switzerland) and primers specific to a 16S region (Eurogentec, Liège, Belgium). Bacteroides species (an abundant species found in the gut) DNA was quantified using a commercially available double-dye probe and primer kit for all Bacteroides species, targeted to a 16S specific region common to Bacteroides species only (Path-Bacteroides_spp, Genesig, Primerdesign Ltd, Chandler’s Ford, UK). Results are presented as Bacteroides/total bacterial DNA ratio.

### Statistical analysis

All statistical analyses of data were performed using the Statistical Package for Social Sciences (SPSS for Windows, version 21.0, IBM, New York, USA). All data are presented as mean values ± standard deviation (SD) unless otherwise stated. Data were checked for normal distribution by observing the *P* value from the Shapiro–Wilk test. Data not normally distributed (absolute plasma I-FABP, Bacteroides/total bacterial DNA ratio) were normalised by natural log (base-e) transformation prior to analysis. A two-way repeated measures ANOVA (arm × time) was performed to compare temporal responses between each arm (Plac and Col) for plasma volume change, HR, RPE, thermal sensation, plasma glucose, plasma lactate, urine volume, urine osmolality, core temperature, total leukocytes, neutrophils, total lymphocytes, neutrophils to total lymphocytes ratio (NEU/LYM), monocytes, absolute plasma I-FABP, percent change plasma I-FABP and Bacteroides/total bacterial DNA ratio. When a main interaction effect was evident, a post hoc paired samples *t* test with Holm–Bonferroni correction was performed on the change (for time) between arms (Plac and Col). Effect size (ES) was calculated using Cohen’s *d* for the Post-Ex and 1 h Post-Ex difference from Pre-Ex between arms (Plac and Col) for plasma I-FABP (absolute and percent change), when a main interaction was evident. To determine any associations, Post-Ex values for absolute plasma I-FABP and Bacteroides/total bacterial DNA ratio were assessed using Pearson’s correlation. It is not possible to pool the data for a single analysis as the repeated measures design (each participant undertook both Plac and Col arms) means this would violate the assumption that observations are independent (required for correlation analysis). As such, each main visit was analysed separately for correlations. We chose to analyse the participants’ visit 1 and visit 2 separately (consistent with previous analysis with similar data by our group [2]) to ensure the necessary independence of observations in each analysis but also ensuring that each analysis contained a mixture of Plac and Col conditions, and hence gave a good representation of the true spread of data for each measure within the study. Statistical significance was accepted at *P* < 0.05 level.

## Results

### Physiological variables and hydration status

One participant completed 56 min of the 60 min protocol in both trials due to core temperature reaching termination criteria (40 °C), but as this occurred at the same point in both trials (and because it was so close to the full 60 min duration) they were still included in the final data analysis. The response of physiological variables (HR, RPE, thermal sensation, plasma glucose, plasma lactate, urine volume, urine osmolality) to exercise was similar between arms (Col and Plac) (Table 1). A similar pattern of plasma volume change was observed from Pre-Ex between arms: Plac; Post-Ex [− 12.6 ± 2.7]; 1 h Post-Ex [− 6.2 ± 2.8] and Col; Post-Ex [− 13.7 ± 1.7]; 1 h Post-Ex [− 6.6 ± 2.5], with no significant difference between arms (*P* = 0.552), as a result no parameters were corrected for plasma volume changes.

**Table 1:** Physiological, hydration status, and perceptual responses to exercise trials

	Pre-Ex	10 min	20 min	30 min	40 min	50 min	60 min	Post-Ex 1 h	ANOVA *P* values (arm; time; arm × time)
HR (bpm)		*	*	*	*	*	*		
Plac	70 ± 12	152 ± 16	163 ± 16	169 ± 16	176 ± 16	180 ± 15	181 ± 14		0.425; <0.0001; 0.095
Col	67 ± 11	155 ± 15	161 ± 14	169 ± 14	177 ± 13	180 ± 12	184 ± 10		
RPE			*	*	*	*	*		
Plac		10 ± 2	11 ± 1	12 ± 1	13 ± 1	15 ± 2	15 ± 2		0.210; < 0.0001; 0.806
Col		10 ± 2	11 ± 2	13 ± 1	14 ± 2	15 ± 2	16 ± 2		
Thermal sensation			*	*	*	*	*		
Plac		2 ± 1	3 ± 1	4 ± 1	5 ± 1	6 ± 1	6 ± 2		0.370; < 0.0001; 0.416
Col		2 ± 1	3 ± 1	4 ± 1	5 ± 1	6 ± 1	6 ± 1		
Plasma glucose (mmol^−1^)							*		
Plac	5.06 ± 0.40						5.95 ± 0.59	4.97 ± 0.38	0.663; < 0.0001; 0.957
Col	5.08 ± 0.48						5.92 ± 0.60	4.95 ± 0.32	
Plasma lactate (mmol^−1^)							*		
Plac	1.14 ± 0.27						3.28 ± 1.22	1.22 ± 0.27	0.619; < 0.0001; 0.206
Col	1.08 ± 0.24						3.35 ± 1.42	1.05 ± 0.12	
Urine volume (mL)								*	
Plac	157 ± 119							70 ± 57	0.700; 0.009; 0.304
Col	144 ± 112							82 ± 51	
Urine osmolality (mOsm kg^−1^)									
Plac	512 ± 276							592 ± 270	0.660; 0.214; 0.588
Col	537 ± 320							635 ± 281	

Post hoc comparisons for main effect of time (overall owing to no interaction effect): *significantly increased (*P* < 0.01) compared to 1st measured time point (Pre-Ex or 10 min)

### Immune cell counts

The response of immune cell counts was similar between arms (Table 2).

**Table 2:** Immune cell count responses to exercise trials

	Pre-Ex	Post-Ex	Post-Ex 1 h	*P* values (arm; time; arm x time)
Total leukocytes (cells × 10^9^ L^−1^)		*	*	
Plac	5.60 ± 1.12	8.51 ± 2.00	8.47 ± 3.37	0.664; 0.003; 0.758
Col	5.59 ± 1.10	8.36 ± 2.07	8.16 ± 3.19	
Neutrophils (cells × 10^9^ L^− 1^)		*	*	
Plac	2.76 ± 0.78	4.64 ± 1.84	5.95 ± 3.10	0.470; 0.002; 0.950
Col	2.62 ± 0.64	4.46 ± 1.83	5.69 ± 2.98	
Total lymphocytes (cells × 10^9^ L^−1^)		*	*	
Plac	2.09 ± 0.43	3.06 ± 0.73	1.69 ± 0.41	0.596; <0.001; 0.305
Col	2.21 ± 0.59	3.10 ± 0.78	1.70 ± 0.49	
Neutrophils/lymphocytes			*	
Plac	1.35 ± 0.38	1.62 ± 0.88	3.67 ± 1.95	0.423; <0.001; 0.990
Col	1.24 ± 0.40	1.53 ± 0.74	3.58 ± 1.93	
Monocytes (cells × 10^9^ L^−1^)				
Plac	0.52 ± 0.13	0.58 ± 0.16	0.58 ± 0.22	0.603; 0.526; 0.440
Col	0.53 ± 0.13	0.56 ± 0.13	0.54 ± 0.13	

Post hoc comparisons for main effect of time (overall owing to no interaction effect): *significantly different (*P* < 0.05) compared to Pre-Ex

### Core temperature

The increase for core temperature was similar in Plac and Col arms (ANOVA effect of arm *P* = 0.997; effect of time *P* < 0.001; and arm × time interaction *P* = 0.473). The mean increase for core temperature was 2.6 ± 0.5 and 2.4 ± 0.6 °C for Plac and Col arms, respectively (Plac 36.8 ± 0.4 to 39.3 ± 0.6 °C; Col 36.8 ± 0.4 to 39.3 ± 0.7 °C).

### Plasma I-FABP

**Fig. 2:**
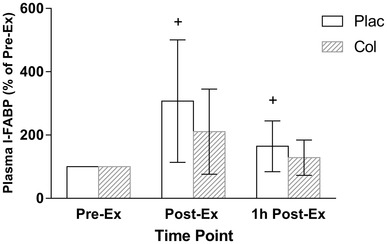
Plasma I-FABP concentration (% of Pre-Ex). +Increase above Pre-Ex significantly greater compared to Col for Post-Ex (*P* = 0.015), and 1 h Post-Ex (*P* = 0.019)

For absolute plasma I-FABP concentration, there was no main effect of arm (*P* = 0.605). There was a significant main effect of time (*P* < 0.0001), and arm × time interaction effect (*P* = 0.005). The Pre-Ex concentrations were similar between Plac and Col arms (*P* = 0.085). Post hoc analysis showed that absolute plasma I-FABP concentration [median and (interquartile range)] significantly increased from Pre-Ex [661 (571) pg/mL] to Post-Ex [1924 (1394) pg/mL] (*P* = 0.01, ES 0.36), and remained elevated at 1 h Post-Ex [993 (598) pg/mL] (*P* = 0.036, ES 0.39) in the Plac arm to a greater extent compared to Col, Pre-Ex [727 (682)], Post-Ex [1781 (1603)], 1 h Post-Ex [993 (598)]. When analysed for percent change, there was a main effect of arm (*P* = 0.011), time (*P* = 0.03), and an arm × time interaction effect (*P* = 0.017). Post hoc analysis showed that percent change in plasma I-FABP concentration significantly increased at Post-Ex (407 ± 194%, *P* = 0.015, ES 0.6) and 1 h Post-Ex (265 ± 80%, *P* = 0.019, ES 0.55) in the Plac arm to a greater extent compared to Col, Post-Ex (311 ± 134%), 1 h Post-Ex (229 ± 56%) (Fig. 2).

### Bacteroides/total 16S rDNA

**Fig. 3:**
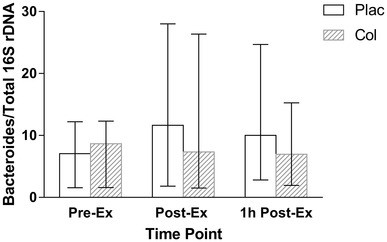
Bacteroides/total 16S rDNA concentration (median and interquartile range). Main effect of arm (*P* = 0.046), but no effect of time (*P* = 0.078), or arm x time interaction effect (*P* = 0.904)

For plasma Bacteroides/total 16S rDNA there was a main effect of arm (*P* = 0.046), but no effect of time (*P* = 0.078), or arm × time interaction (*P* = 0.904). Pre-Ex values were similar between Plac and Col arms (*P* = 0.540). Bacteroides/total 16S rDNA concentrations [median and (interquartile range)] were 2.76 [3.54] × 10^−4^ at Pre-Ex, 4.55 [8.96] × 10^−4^ at Post-Ex, and 3.91 [6.24] × 10^−4^ at 1 h Post-Ex, and 3.39 [4.02] × 10^−4^ at Pre-Ex, 2.87 [8.94] × 10^−4^ at Post-Ex, and 2.72 [3.81) × 10^−4^ at 1 h Post-Ex, for the Plac and Col arms, respectively (Fig. 3).

### Association between absolute plasma I-FABP and Bacteroides/total 16S rDNA

**Fig. 4:**
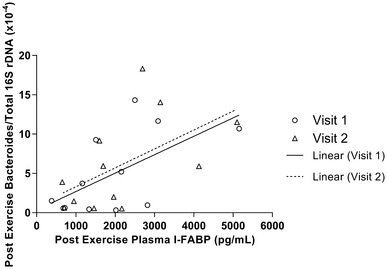
Association between post-exercise absolute plasma I-FABP and Bacteroides/total bacterial DNA. Visit 1 (*P* = 0.038, *R*_s_ = 0.603) and Visit 2 (*P* = 0.060, *R*_s_ = 0.557)

The Pearson’s correlation analysis showed that Post-Ex absolute plasma I-FABP concentration significantly correlated with the Post-Ex Bacteroides/total 16S rDNA for Visit 1 (*P* = 0.038, *R*_s_ = 0.603) but not Visit 2 (*P* = 0.060, *R*_s_ = 0.557), although plots do suggest similar overall patterns (see Fig. 4).

## Discussion

The main findings of this study are that (1) plasma I-FABP increased when running at 70% ${\dot {\text{V}}}{\text{O}_{2\text{peak}}}$ in the heat for 60 min, (2) 14 days of oral Col supplementation was able to blunt the increase in plasma I-FABP, but did not influence the exercise response of plasma Bacteroides/total 16S rDNA (although there was a main effect of arm). This is the first study to demonstrate the efficacy of Col in vivo on intestinal injury in exercising humans within a heat and exercise stress model and expands on our previous work showing the benefit of Col on the intestines following exercise [1, 2, 11].

The increase in plasma I-FABP would suggest that intestinal barrier integrity in the current study was compromised as changes in plasma I-FABP have previously been shown to be associated with increases in permeability [2]. Furthermore, these increases in plasma I-FABP following exercise were to a greater extent than observed in our previous study during thermoneutral conditions [2], and may be associated with changes in circulating Bacteroides/total 16S rDNA. The 407 ± 194% increase in plasma I-FABP was over double the increase we have observed previously [2], where we reported a concurrent increase in intestinal permeability as measured by urinary L/R (5 h). Previous investigations reporting either significant increases in core temperature or an end core temperature > 39 °C (or both as seen in the present study) with treadmill running observed significant changes in permeability as measured by urinary L/R (5 h) [2, 3, 11, 34]. A recent study has suggested that when heat stress induces increase in core temperature to greater than 39 °C then Col may not be efficacious [15]. However this study [15] had significant limitations: they only reported a 162% increase in plasma I-FABP despite exercising at higher ambient temperature than the current study (40 vs 30 °C), which was also reflected in a marginal greater rise in core temperature. Furthermore, the increase (162%) in I-FABP previously reported [15] is one of the lowest reported in the literature, lower than reported increases following high intensity interval training (172%) [32], following exercise in the heat (168%) [35], and following cycling in a controlled thermoneutral condition (172%) [31]. This relatively low post-exercise increase in plasma I-FABP may explain their lack of reported effect [15] of Col supplementation. Moreover, McKenna et al. [15] reported high pre-exercise levels of plasma I-FABP which may also offer an explanation, and finally but perhaps most significantly, there were variable running times between the two conditions, imposing different stresses between conditions (placebo and Col), which likely confound their results.

Running in the heat causes a greater redistribution of blood flow towards the cutaneous region to dissipate heat resulting in greater hypoperfusion, and ischaemia in the intestines than exercising in temperate conditions [36]. The increase in plasma I-FABP during exercise appears to be as a result of increases in core temperature, hypoperfusion and I-R [2, 5, 31]. Both comparable physiological temperatures (and lower) as reported in the present study, and hypoperfusion and subsequent ischaemia result in tight junction (TJ) breakdown [37, 38]. Col has been previously shown to blunt the in vivo increase in intestinal permeability in animals heated to a core temperature of 41.5 °C [10]. The mechanism for the efficacy of Col is through an up-regulation of TJ proteins (such as Claudin-1, Claudin-2 and zonula occluden-1 protein), and its ability to induce favourable changes in caspase-3, caspase-9, Baxα and Bcl-2 as has been demonstrated in vitro in human cell lines [1, 11, 39]. Therefore, Col is able to upregulate TJ proteins, maintaining cell to cell contact (and thus cellular integrity under stress) preventing cellular damage, as indicated by a blunting of plasma I-FABP in the Col arm. Moreover, direct protective cellular effects (e.g. up-regulation of heat shock protein [HSP]) prompted by Col, which has previously been demonstrated [1, 11], may also blunt the increase in plasma I-FABP.

Recent studies have shown that significant rises in core temperature [measured at the rectum and oesophagus (approx. 1.5–2 °C)] during exercise in the heat result in significant elevations in plasma I-FABP [2, 15, 40]. The larger increase in core temperature (measured at the rectum) in the present study (~ 2.5 °C) in comparison with our previous work (1.5 °C increase) [2], was also reflected by greater increases in plasma I-FABP. Not only does this highlight the stress that both exercise and heat place on the intestine (in accordance with changes in circulating I-FABP) but furthermore shows that this dose and timing (20 g day^−1^ for 14 days) of Col supplementation are only able to partly blunt intestinal injury during exercise with combined heat stress. This finding is in agreement with our results from a series of previous investigations [1, 2, 11] showing that this supplementation regime cannot fully blunt the increase in intestinal damage during exercise. This also supports the notion that combinations of supplements to confer further benefit against intestinal damage in the exercise (and heat) stress model may be more efficacious [1].

Consistent with previous studies [5, 41], plasma I-FABP levels approximated pre-exercise levels in the current study 60 min following the termination of exercise which likely underlines the ability of the intestines to withstand short periods of reduced perfusion (ischaemia) and to rapidly restore the intestinal barrier (restitution). Full restoration of the intestinal barrier through intestinal cell migration 60 min after a 30 min ischaemic period has previously been reported [42]. However, even these comparatively short periods of intestinal barrier injury (where permeability is increased) are likely still of clinical relevance. For example, LPS translocation has previously been described following exercise of shorter duration than the present study [43]. It is possible that Col could enhance the migration of intestinal cells in this post-ischaemic period which has been previously shown in vitro [44].

In the present study, and previously we have shown that Col supplementation can blunt both the exercise-induced increase in intestinal permeability as measured by the urinary L/R (5 h) [1, 2, 11] and cellular damage as indicated by plasma I-FABP [2]. However, it may be considered more pertinent to investigate the effect of Col supplementation on the consequence of this increase in permeability (i.e. bacterial translocation) as this is associated with endotoxaemia and in some cases (although rare) severe outcomes. Although the aforementioned issues pertaining to the collection and analysis of blood samples, in addition to the detection of LPS (some components of human plasma can interfere with the LAL assay resulting in false positives), suggest that the use of this marker to assess the impact of intestinal permeability changes is less than ideal [18, 19]. Therefore, in the present study we measured the ratio of Baceteroides to total bacterial DNA. Bacteroides account for around 25% of the anaerobes found in the gastrointestinal tract, and are the most prevalent bacteria in the gut [45]. In the present study, Bacteroides/total 16S rDNA appeared to be lower overall following 14 days of Col supplementation which may indicate a direct effect of Col on intestinal permeability and subsequent bacterial translocation but it must be noted that there was no arm × time interaction showing the overall response to exercise was not influenced by Col. There was large inter-participant variation in this measure, and whilst this method appears advantageous in comparison with LPS assays (e.g. LAL) due to specificity for a particular bacterial strain, it may require a larger sample size to detect the magnitude of effect observed in this study. Alternatively, it may be valuable to also measure other abundant gut bacterial species at the same time to further explore the effects of exercise and Col. This is the first study to assess the effect of Col supplementation in an exercise and heat stress model with measures of bacterial translocation using the nucleic acid testing (NAT) model for bacterial DNA. It provides further evidence that 14 days of Col supplementation can reduce the increase in exercise-induced intestinal permeability, and preliminary evidence that this may reduce bacterial translocation, but the latter requires further research (i.e. with a larger sample size and/or adding other bacterial strains to the detection panel). This study and others [2, 30–32] have observed a large inter-participant variability (Plasma I-FABP was highly variable in both arms) in plasma I-FABP; therefore, future investigations should endeavour to understand how plasma I-FABP is influenced by factors such as diurnal variation and how it changes in the post-prandial period.

A limitation of the present study is that we did not attain training history or current training status from participants, it has been speculated that regular training may be protective against exercise-induced changes in intestinal permeability through an up-regulation in HSP [22, 46]. However, this has yet to be reliably demonstrated [47], and furthermore in the current study there was little variation for ${\dot {\text{V}}}{\text{O}_{2\text{peak}}}$ values (SD < 5 mL kg^−1^ min^−1^) indicating a similar level of aerobic fitness for participants in the study (whom were all regular exercisers). Finally, the protocol employed in this study required participants to exercise at the same relative intensity, therefore, imposing a similar physiological stress between participants despite small variances in fitness through training status.

## Conclusion

This is the first study to demonstrate the efficacy of Col to reduce intestinal injury following exercise in the heat. This could hold particular relevance to athletes who are required to compete in hot and humid conditions and those individuals whose work (e.g. soldiers, firefighters) necessitates exhaustive physical exertion in such environments. Future studies may wish to determine the effects of Col in clinically diseased populations where endotoxaemia and subsequent inflammation contribute to the pathophysiology of the condition.

## Abbreviations

ANOVA: Analysis of variance
Col: Bovine colostrum
DNA: Deoxyribonucleic acid
Hb: Haemoglobin
HR: Heart rate
HSP: Heat shock protein
I-FABP: Intestinal fatty acid-binding protein
I-R: Ischaemia–reperfusion
LAL: Limulus amoebocyte lysate assay
LPS: Lipopolysaccharide
NAT: Nucleic acid testing
Plac: Placebo
PCR: Polymerase chain reaction
RPE: Rating of perceived exertion
L/R: Urinary excretion ratio between lactulose and rhamnose
TJ: Tight junction
